# Coalition Formation Game for Cost-Efficient Multiparty Payment Channel in Payment Channel Networks

**DOI:** 10.3390/s23094524

**Published:** 2023-05-06

**Authors:** Wooseong Kim

**Affiliations:** Computer Engineering Department, Gachon University, Seongnam-si 13120, Republic of Korea; wooseong@gachon.ac.kr

**Keywords:** blockchain, cryptocurrency, payment network, multiparty payment channel, coalition formation game

## Abstract

Blockchain has introduced a new era for online payment services and its economy with tamper-proof cryptocurrencies. However, blockchain, which is based on global peer-to-peer networks, has its limitations due to payment delays from global consensus and transaction costs for maintenance. Thus, payment channel networks (PCN) have been proposed as one of the most promising off-chain solutions, allowing users to pay directly through payment channels (PC), with minimal blockchain involvement. However, payment delays and cost problems still exist, especially given the large size of the PCN. This study proposes a multiparty payment channel (MPC) that enables multiple users to join the same PC and exchange payment transactions, compared to the legacy PC. To avoid a consensus procedure among users in the PC, we introduce sequential and parallel updates for the PC status. Since increasing the MPC size limits the advantages in terms of the delay and cost, we propose a distributed coalition formation algorithm to form the MPC group, in which each user has the choice to join or leave the group. Simulations show that the proposed algorithm establishes MPCs successfully, considering the trade-off between the payoff gain and the MPC delay cost.

## 1. Introduction

With the many successes of cryptocurrency, the blockchain has been confirmed as a tamper-proof ledger operating in autonomous peer-to-peer (P2P) networks. However, the blockchain suffers from inherent scalability problems as the number of nodes and transactions grows rapidly. The consensus algorithms, such as proof of work for Bitcoin [1] and Ethereum [2], require excessive time to order transactions chronologically and synchronize them globally. Although many consensus algorithms, termed PoX (e.g., Proof of Stake [3], Delegated Proof of Stake [4], Proof of Validation [5], Proof of Elapsed time [6]), have been proposed to improve the performance in terms of transactions per second, it is still critical to maintain a unique chain agreed by globally distributed nodes.

Instead, multiple leading chains can be used to deal with transactions in parallel, for which separate committees of these chains decide the final status of their chain independently, called blockchain sharding (e.g., RScoin [7], omniLedger [8], or rapidChain [9]). Similarly, parasite chains (called side chains) plugged into the main chain can be used to improve scalability, e.g., Plasma [10]. However, such multiple-chain approaches require periodic mutual verification for integrity checks. In contrast to the legacy blockchain, a directed acyclic graph (DAG)-based blockchain (e.g., IOTA [11], Nxt, ByteBall, and coDAG [12,13,14]) allows forking, which enables asynchronous updates within multiple side chains, converging in a specific direction. It is vulnerable to double spending by split attacks or long parasite chaining and still relies on a global consensus.

In this paper, we focus on the L2 protocol for the blockchain network, called an “off-chain” protocol [15], by which two users can create a local ledger (“payment channel (PC)”) for transactions between them and pay each other directly by updating the balance information in the PC [16]. Therefore, the off-chain technique completes transactions faster than the blockchain without a consensus and fee.

The PC is expanded to payment networks for indirect payment, called payment channel networks (PCN), because all users cannot have direct PCs to each other. A user can pay someone indirectly through multiple PCs of unknown intermediary users and payment service providers (PSP), such as multihop routing in ad hoc networks. Such multihop payment is a path-based transaction (PBT) in the PCN. This PCN is a promising technology to improve the scalability and efficiency of the main blockchain. Several off-chain payment networks have been proposed and developed for cryptocurrencies, such as the Lightning network [17] for Bitcoin, Raiden network [18] for Ethereum, and Paymo [19] for Monero.

There have been many studies about the PCN. First, the PBT routing algorithm was explored, which is critical to protect privacy and maximize the PCN throughput. Flare [20], as the first routing protocol, finds a feasible transaction path in the PCN by mimicking a multihop ad hoc network protocol for the Lightning network. SilentWhispers [21] and SpeedMurmurs [22] introduced efficient decentralized routing algorithms for fast and private payment settlement in the PBT. VOUTE [23] followed, enhancing the efficiency of the landmark routing of SilentWhispers. Meanwhile, Rayo and Fulgor [24] provided privacy and concurrency mechanisms in the PBT. Several studies proposed ideas to improve the PCN performance, limited by the channel deposit amount and PBT length. Spider [25,26] and Flash [27] introduced atomic multipath payments, which increased the PCN throughput by splitting a large PBT flow into many small flows. A redundant and secure balance was planned [28,29] and transaction forwarding was scheduled [30] by the mathematical optimization model, assuming that the payment demands are known. Some studies investigated the economic aspects of the PCN [31,32]. Cheapay [33] and payGo [34] found an economic payment path in terms of low fees of PSPs.

In this study, we focus on the two major challenges of the PCN as below.

- Unbalanced collateral: The PC user balance varies dynamically with payment flows. Accordingly, asymmetric payment flows cause unbalanced collateral and balance depletion, which stops payment for upcoming PBTs.
- Collateral cost: A PSP must lock the collateral amount for each PBT to prevent double spending. This collateral cost increases with the number of PBTs and their delays.

First, the exhaustion of the balance due to the biased payment direction has been solved by an artificial opposite PBT flow. Revive [35] and Circle [36] search the circular payment flows to rebalance the channel. However, this approach assumes mutual trust among users on the circular path and has difficulty in finding the circular path without causing an imbalance in other channels. Chen et al. [37] introduced a MPC for rebalance that provides the order of transactions among multiple PCs within a group. This work does not consider utility for MPC formation; accordingly, a given MPC topology limits rebalance. Second, there are several L3 approaches to rebalance deposits among existing L2 PCs. Burchert et al. [38] introduced the channel factory concept that reassigns deposit tokens to a specific pairwise channel dynamically based on agreement by *n*-party users. Dziembowski et al. [39,40] proposed the virtual channel concept that creates a direct virtual channel between two users linked by multiple PCs. Following this, more studies [41,42] improved upon the virtual channel concept. However, the security of these concepts relies on the behavior of underlying PSPs.

This study proposes a multiparty payment channel (MPC) only using the L2 protocol that reduces the collateral cost of the PSP and the probability of deposit imbalance. For instance, if four users with pairwise channels are grouped into an MPC, the collateral cost decreases by one third, while the probability of incoming PBT flows increases by three. The MPC adopts slotted payment for sequential updates of an off-chain state. However, the utility of a grand coalition does not increase additively due to the delay cost in underlying networks. Because the turnaround delay for individual payment slots increases proportionally to the MPC size, multiple MPC coalitions must be created regionally to maximize their utility in the PCN. We propose a distributed algorithm based on the switching operation for each PSP in such a coalition formation game, which permits the PCN to converge to the Nash equilibrium of MPC partitions. We simulate the user payment flows in random social networks and investigate the performance of the MPC formation algorithm with varying turnaround delays and cost parameters. Experimental results show that the proposed algorithm forms MPC coalitions stably and improves the PCN utility consistently by limiting the individual MPC size according to the incidental cost.

In summary, our key contributions in this study are highlighted below.

- A multiparty payment channel is designed above Layer 2 in the blockchain network.
- Expected threats to the MPC are discussed, such as double spending and the Sybil attack.
- A mathematical model is established to derive the optimal MPC size.
- A distributed MPC formation algorithm is proposed for this coalition formation game.

The rest of this study is organized as follows. First, the background of the PC and network is briefly reviewed in Section 3. We introduce the MPC algorithm and its protocol design with smart contracts in Section 4. Section 5 describes a distributed MPC formation algorithm that maximizes PSP utility in the PCN. In Section 6, we evaluate the performance of the coalition formation algorithm and discuss the experimental results. We conclude this paper in Section 7.

## 2. Related Works

The payment channel imbalance and depletion problem is a well-known problem and has been studied for the past several years. In this section, we introduce details of these studies and compare them as in Table 1. Since the channel depletion is caused by imbalanced payment flows in the PCN, many researchers solved it by equalizing the PBT flows. As a seminal work, Revive [35] proposed an artificial opposite PBT flow to achieve balance between bi-directional PBT flows within a payment channel. For this, a user has to find a circular path for the rebalancing, in which each user probably suffers from imbalanced flows. Revive has a critical limitation in that all users along the circular path should trust each other; otherwise, there is no mechanism to receive payback when a certain party denies pay forwarding. For this, Circle [36] was recently proposed, which creates a smart contract to prevent the misbehavior of these participants in the circular payment.

In [38], a multiparty channel was proposed for the first time for the Bitcoin micro-payment channel. The authors created a middle layer between the Bitcoin and micro-payment channel layers, called a channel factory, that replaces a transaction with a shared lock on the channel deposit whenever a rebalance among the micro-payment channels is required. However, it is still costly for relocation transactions due to the nature of the Bitcoin network and relies on complicated data structures of timelocked transactions for state consensus.

Perun, as a virtual payment channel over the L2 protocol, was proposed in [39,40]. Perun is a payment hub that has direct channels with several parties without intermediary users. The authors extended Perun for a general multiparty channel [41]. Perun can be easily realized by smart contracts, compared to previous studies. However, it also assumes that the intermediary users along the virtual channel are trusted or can be punished for misbehavior by the smart contract. Accordingly, Perun can reuse the deposit for the virtual channel but it requires smart contracting with a delay. Meanwhile, [42,43] extended Perun to the Bitcoin network as a lightweight virtual payment channel, only requiring timelocks and multi-signatures.

Recently, the MPC concept based on multiple PCs was introduced in [37], which is designed only for a fixed PCN topology, in contrast to our proposal. As the MPC demands consensus on every transaction to all parties and schedules its individual PCs proportionally to balance, the transaction latency can increase exponentially according to the number of users. However, MPC formation is not discussed for such a scalability problem.

## 3. Background

In this section, we introduce the PC and PCN briefly as a background and discuss channel imbalance as a key challenge of the PCN. The PC is a logical connection between two users who exchange messages for payment, improving the transaction scalability due to the absence of blockchain involvement. For this, the users open the PC, a type of escrow account, by depositing an amount using a smart contract, executing over the public Ethereum network, and the balance amount cannot be withdrawn without the agreement of the other party; there is an alternative approach for the Bitcoin network. After this, these two users pay each other safely within their own balance.

The PCs can constitute a network, a PCN, as a communication link through the Internet, in which users are able to pay someone remotely, without a direct PC. Such a remote payment, called a PBT, can be relayed through multiple intermediary PCs of unknown users along a payment path that is discovered by a routing protocol for the PCN [34]. Consequently, the PBT mechanism of the PCN can solve the scalability problem expected with countless users in the off-chain model. In the following subsections, more detailed procedures are explained.

### 3.1. Direct Payment Channel

The PC is a local ledger recording transactions between two users, in which they can update the ledger without blockchain intervention. In other words, consensus on the channel state is made only by the two users who agree with the new balance of the PC; a user verifies the signature of the counterparty on the new transaction and adds their signature.

The main blockchain governs only the opening or closing of the PC. To open the PC, two users deposit an amount in an escrow account through the smart contract to prevent double spending. If he disagrees on the PC state, the user ignores it and asks to close the channel through the smart contract. Then, the smart contract returns the amount based on the last PC state. Since these two procedures require transaction fees in the main blockchain, they will not invoke the unnecessary closing procedure.

This study explains the MPC mechanism using a bidirectional PC created by the smart contract in the Ethereum network. Any other PCs, such as a micro-PC for Bitcoin [44], can also be used for the MPC.

### 3.2. Multihop Payment Channel Network

**Figure 1:**
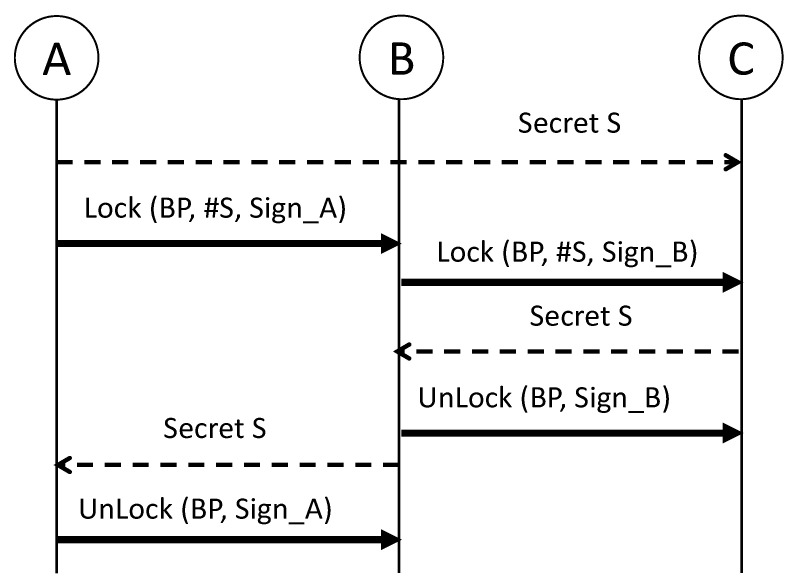
A PBT from the user *A* to the user *C* via the user *B*. For the atomic operation of the PBT, each user pays conditionally by locking the amount with a new conditional BP. Later, the user settles the payment by unlocking with a new complete BP after receiving the proof of payment reception, the pre-image (*S*) of a hash value #S.

It is costly and impractical for a user to create a PC for a new payee every time. The PCN supports indirect payment through multiple PCs based on the conditional balance proof (BP) concept of Ethereum that guarantees atomic payment between a payer and payee, which works similarly with the hashed timelocked contract of the Lightning network [17]. Details of this protocol are described in Figure 1.

For the PBT procedure from the user *A* to *C*, the user *A* sends a conditional BP, including a new channel balance with his signature, to the user *B*, preventing double spending by *A*. The conditional BP can be valid only when the receiver secures a pre-image of the hashed secret #S included in the BP. The receiver can reimburse the amount of the conditional BP from a counterparty or smart contract in the main chain if the counterparty does not unlock it after receiving the *S* until the deadline. The smart contract verifies the pre-image *S* and hash value, #S = SHA256 (*S*), for the balance update, returns the amount based on the final balance, and closes the PC. Accordingly, rational users behave honestly to keep the channels open for future transactions or monetary incentives from PBT relaying [34]. The receiver can try to request the unlock repeatedly from the counterparty, rather than the arbitration of a smart contract for dispute resolution.

The Raiden network [18] supports concurrent PBTs (i.e., PBT flow) with a Merkle tree of information of the in-flight conditional payments, such as the amount, a lock timer, a secret hash, the public key of a payer, etc. Concurrency is also supported in the Bitcoin-compatible PCN [45]. To avoid a balance deficiency due to many in-flight PBTs, each conditional payment has a lock timer for a deadline that prevents collateral from being locked infinitely. Expired conditional BPs are discarded, and the corresponding amount can be used for the next payment if no PSP along the payment path unlocks before the PBT is canceled. However, finding an optimal lock time is challenging based on previous PBTs; the tight duration can cause frequent payment failures and channel closures, while a loose duration increases the collateral costs.

### 3.3. Payment Channel Imbalance

**Figure 2:**
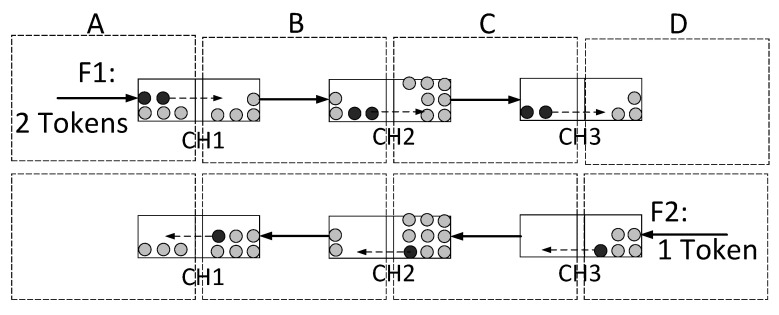
Two flows of path-based transactions (PBTs), F1 and F2, in opposite directions, have different token rates and cause channel imbalance. For example, the user *B* will have no token in the CH2 after more than two seconds.

Asymmetric PBT flows can cause a channel imbalance that induces a balance deficiency and degrades the payment throughput in the PCN. Figure 2 depicts the dynamics of tokens at three channels with two asymmetric PBT flows F1 and F2. The PBT F1 moves two tokens toward the counterparty every second, while F2 moves backward only one token. This asymmetric PBT flow eventually causes balance depletion for one of the channel users.

We investigate the PCN performance with the imbalanced PBT flows as in Figure 2 using a discrete-time PCN simulator [34]. For the experiment, unlimited tokens are given to the payers, *A* and *D*, while the other two PSPs, *B* and *C*, have fixed deposits. Each payment amount of the PBTs is configured as 50% of the initial channel deposit. The message propagation and processing delay follow an exponential distribution of 10 and 1 ms, respectively. The time lock is set as 3 s for each transaction.

**Figure 3:**
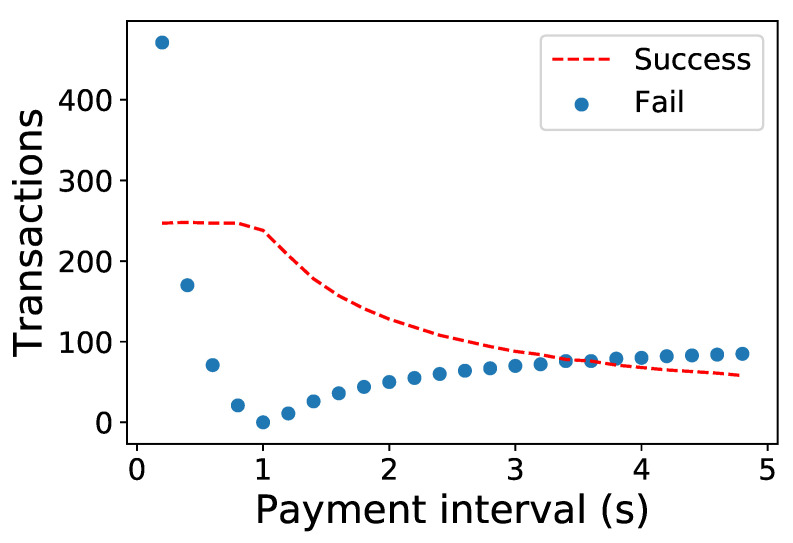
Number of successful and failed transactions in a linear PCN. The PBT interval of the F1 varies while the F2 is fixed as 1 s.

Figure 3 shows the success and failure probability of payment, which depends on the balance of the PBT flows. Asymmetric flows by different payment intervals cause significant transaction failures significantly below 1 s due to a higher PBT rate of F1. Meanwhile, symmetric flows with 1 s have no failed transactions. The number of successful transactions as throughput is limited by a flow with a lower PBT rate, which decreases as the F1 rate diminishes, as shown in the figure.

Consequently, the PC performance relies on the channel balance, which is dynamically changed with the PBT flows $x_{ij}$. Furthermore, the channel deposits should be larger than the amount of each PBT, even for balanced PBT flows. We propose an MPC solution that maximizes the throughput and minimizes payment delay under unbalanced PBT flows and limited channel deposits.

## 4. Multiparty Payment Channel

In this study, we propose a novel MPC that consists of multiple PSPs in distributed PCNs. Each PSP user can have multiple PCs to other PSPs only with a single deposit, which increases the collateral efficiency compared to the conventional PCN. For instance, the MPC architecture enables the collateral cost of the legacy PC to increase quadratically $I^{2}C$ with users I and channel deposit *C*, and linearly IC in the MPC. Additionally, the MPC can mitigate channel imbalance or depletion via asymmetric PBT flows since an MPC user has more incoming PBT flows from N-1 peers.

In contrast to previous MPC approaches based on the existing PCs, our MPC is based on the consensus of all PSP members for a ledger update. The MPC can have multiple transactions concurrently occurring between two particular parties, which needs to be ultimately acknowledged by all members. Accordingly, the update order should be given to avoid ambiguity of the MPC state and prevent subsequent misbehavior such as double spending. For this, we assign a numerical identifier to each virtual sub-channel between two PSPs, which provides order information of the MPC state and traceability in the MPC balance. We explain the details of the MPC mechanism from channel creation to channel state update in the following subsections, with the symbols in Table 2.

### 4.1. MPC Creation

As addressed in our previous study [34], where a PC is created by an L2 protocol and smart contract running in an Ethereum virtual machine, the MPC can be created similarly by multiple users. Users execute individually a registration function to open a channel with information parameters, e.g., an initial deposit, an account address, a host IP address, etc. To close the channel, a close function is called with a final channel state and the signatures of all users, which returns the amount to each user based on the final channel balance. The final MPC state is determined by the transaction with the highest round count, which increases for every transaction.

### 4.2. MPC Consensus Algorithm

In contrast to the legacy PC, in which interest is a conflict between two users, the MPC needs a consensus algorithm as users can collude in their own interests against other participants. Majority-based consensus algorithms for the public blockchain are infeasible since securing more than 50% users in such a small group is easy. For the small-sized private blockchain, Byzantine Fault-Tolerant (BFT) protocols, such as Practical BFT (PBFT) [46,47,48], are popularly considered. However, this permission-based approach in which all users are admitted by a certificate authority is inapplicable for the MPC, which is a permissionless off-chain network, as with the legacy PC.

The MPC consensus algorithm has to resolve the following two challenges:

- State update of the MPC, from M(t) to M(t+1).
- Transaction serialization in the local chain of the MPC.

All users should form a consensus for the MPC state update by signing on a new transaction that changes the channel state. This consensus procedure requires heavy peer-to-peer communication that causes a considerable delay, especially with many MPC members, *N*.

A unique round count should be assigned to each transaction sequentially for serialization. Simultaneous payments fork the local chain with multiple transactions having the exact round count. Unfortunately, the MPC cannot resolve the dependency between concurrent transactions based on the majority.

Therefore, we introduce a time-slotted sub-channel or sub-PC (SPC) concept for the MPC. At each time slot denoted by the round count *t*, only two MPC users, called SPC users, can create a new transaction with the given round count.

**Figure 4:**
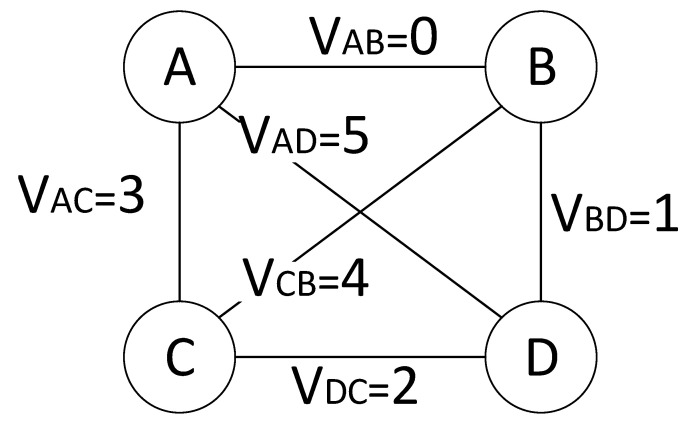
Example of 4 users for a multiparty payment channel.

Figure 4 illustrates an example of the MPC with four users, N = { A, B, C, D }. Maximum N(N−1)/2 bidirectional SPCs can exist in the MPC; each bidirectional SPC is denoted by $v_{ij}=v_{ji}$ for ij∈V,V⊆L. Each SPC has a time slot (i.e., round count) periodically, with the value of $v_{ij}\in \left[0,V-1\right]$, as shown in Figure 4; a time slot *t* is assigned for a SPC $v_{ij}$ if $v_{ij}\equiv t\;\mathrm{mod}\;V$, where and $t\in Z^{+}$. For example, in the figure, payments in the SPC $v_{BD}$ will be conducted only at the time slot $t_{v_{BD}}=\left\{1,7,13,19,\ldots \right\}$. The duration of each time slot varies with the state update delay from message propagation between SPCs.

Once the users of an SPC receive a local chain from anyone of a previous SPC, they must verify the blocks in terms of balance and a round count signed by the previous SPC users to update the MPC state. After this, they add a new block for their transaction to the local chain and send it to the following SPC users. For example, users A and B exchange their BPs (e.g., A: 100, B: 70), including the round count t=0, which are signed by their private keys at $v_{AB}$, which leads to the update of the MPC state as M(t=0). One of the users, A or B, sends the local chain, including the new block, to either of the users B or D. Subsequently, the users B and D verify the MPC state M(t=0) and follow the same procedure for a new state M(t=1). In the event that the received block has incorrect balance or count information, the users can initiate a dispute process with a smart contract in the blockchain, eventually preventing forking and dishonest behavior such as double spending in the MPC.

### 4.3. MPC State Synchronization

#### 4.3.1. Full Synchronization

The MPC state is updated immediately by broadcasting the new transaction block or using a central repository.

- Broadcast: SPC users unicast the new block data directly to all MPC members, (N−2)/2 peers per SPC user. Otherwise, a spanning tree for the MPC is maintained for block propagation.
- Repository: the MPC can designate one of the members as a server for the local chain repository. Once one of the SPC users uploads a new block to the repository, other members can query the update.

The broadcast requires additionally a node discovery and routing protocol to build up a minimum spanning tree, while the repository can suffer from a single point of failure and congestion.

#### 4.3.2. Partial Synchronization

For scalability, the MPC state can be partially synchronized along the order of the SPCs, which allows each user to update the global view only at their turn. For this, a user of an SPC $v_{ij}\left(t\right)$ sends the updated local chain only to users of the next SPC $v_{mn}\left(t+1\right)$, which can therefore reduce the communication overhead compared to full synchronization.

**Figure 5:**
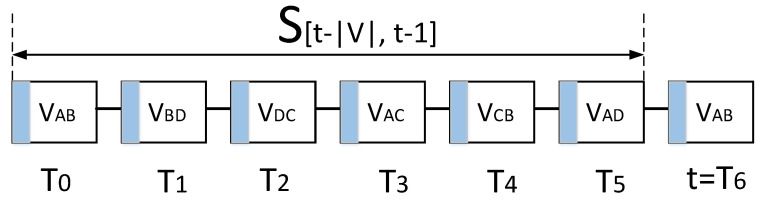
Local chain structure for 4 users and 6 SPCs of the MPC in Figure 4. To verify the final MPC state, user A holds total *V* previous blocks at $t=T_{6}$. Subsequently, the new update by $v_{AB}\left(t=T_{6}\right)$ removes the obsolete $v_{AB}\left(t=T_{0}\right)$.

For partial synchronization, each SPC user verifies *n* previous SPC blocks, $v_{ij}\left(t-n,t\right)$, of the received local chain for the MPC state update if the last state that the user confirmed is $v_{ij}\left(t-n-1\right)$. For example, in Figure 5, user B must confirm the current MPC state before trading with counterparty A at the $T_{6}$. Since the balance of user A could be changed in the block of $v_{AD}$ at the $T_{5}$, user B must acquire and verify the $v_{AD}\left(t=T_{5}\right)$ block. If the $v_{AD}\left(t=T_{5}\right)$ transaction is incorrect, user B can invoke a dispute procedure and close the MPC. Contrarily, user A does not need to update at $T_{6}$ because the last MPC state is already known at $T_{5}$.

Therefore, the number of blocks to verify depends on the SPC schedule as below.

- Periodic SPC for minimum blocks: in fully connected PSPs that individually have N−1 SPCs over total N(N−1)/2, each PSP has its own SPC every N/2 round and the same number of blocks to verify.
- Aperiodic SPC for minimum communication: as shown in Figure 5, SPCs can be linked by an anchoring node that can reduce the communication overhead for local chain propagation. According to the Euler trail algorithm, all SPCs can be visited without additional communication if the *N* is an odd number, and a single extra communication instance is needed otherwise. However, in the worst case, the maximum number of verification blocks can be V−N+1.

The aperiodic SPC probably has a serious update overhead with the propagation of previous *n* blocks if the MPC size, *N*, grows exponentially, even though each block is only around a hundred bytes, containing a round count (4 bytes), user accounts (20 bytes × 2), the BPs of two users (4 bytes × 2), and the two signatures of i,j of $v_{ij}$ (32 bytes × 2). In this study, we use the periodic SPC schedule in the following algorithms.

Algorithm 1 describes the update procedure in the MPC. An SPC user updates the MPC state sequentially along the order of blocks in the received chain unless the signatures are wrong or the balance sum differs from the previous state. Otherwise, the dispute procedure will be triggered by the user. During the dispute procedure, the smart contract settles the balances based on the final MPC state. Algorithm 2 shows the dispute resolution algorithm conducted by the smart contract. All participants upload their own *V* blocks of the last SPC cycle $v_{ij}\left(t\right),t\in \left[n-V,n-1\right]$ before closing the channel, where the last round counts *n* can vary for each user. Subsequently, the smart contract orders receive blocks chronologically and verify each one to update the MPC state sequentially, as in Algorithm 1. In the end, the block with the highest round count finalizes the user balance.
**Algorithm 1:** MPC state update.1:Initialization:2:Set $K_{p}^{i}$, i∈N3:Set last MPC state $S\left(t\right)=\{A_{i}\left(t\right)|i\in N\}$4:Receive blocks $\left\{B_{t}\left(i,j\right)|t\in \left[n,n+\left\lfloor \left(N/2\right)\right\rfloor \right]],i,j\in N\right\}$, where each block $B_{t_{m}}\left(i,j\right)$ has $\left\{A_{i}\left(t_{m}\right),A_{j}\left(t_{m}\right),H,Sign\left(H,K_{s}^{i}\right),Sign\left(H,K_{s}^{j}\right),t_{m}\right\}$5:Instruction:6:**for** $t_{m}\in \left[n,n+\left\lfloor \left(N/2\right)\right\rfloor \right]$**do**7:   **if** $t_{m}==\gamma_{v_{ij}}$ **and**   Verification $Sign\left(H,K_{s}^{i}\right),Sign\left(H,K_{s}^{j}\right)$ by $K_{p}^{i},K_{p}^{j}$ **and**   $A_{i}\left(t_{m}\right)+A_{j}\left(t_{m}\right)$ == $A_{i}\left(t\right)+A_{j}\left(t\right)$ **then**8:     Update MPC state $M\left(t\right)\leftarrow M\left(t_{m}\right)$ with new $A_{i}\left(t_{m}\right),A_{j}\left(t_{m}\right)$9:    **else**10:     Invoke dispute procedure11:   **end if**12:**end for**13:return M(t)

**Algorithm 2:** Smart contract for closure of MPC.
1:Initialization:2:Receive blocks $\left\{B_{t}\left(i,j\right)|t\in \left[n-V,n-1\right],i,j\in N\right\}$ from ∀i∈N3:Order all received $B_{t}\left(i,j\right)$ by the time *t* in the set B4:Instruction:5:**for** $B_{t_{m}}\left(i,j\right)\in B$**do**6:   $M_{ij}\left(t\right)\leftarrow$ MPC state update by $B_{t_{m}}\left(i,j\right)$ in Algorithm 17:   **if** $M_{ij}\left(t\right)$ is valid and $\sum_{i}A_{i}\left(t\right)$== Deposit **then**8:     M(t)←$M_{ij}\left(t\right)$9:    **else**10:     Discard $M_{ij}\left(t\right)$11:   **end if**12:
**end for**
13:Withdraw amount to each node based on M(t)


We discuss the possible security threats in the MPC and their resolutions through the dispute algorithm.

- Double spending: if a malicious user attempts to pay with the wrong BP information, it is detected by a counterparty based on previous blocks, which denies the proposed transaction. Although the counterparty accidentally signs the transaction of the double spending at $T_{n}$, the smart contract can later detect the transaction that contradicts a previous $T_{n-1}$ MPC state.
- Sybil attack: if user B, C, or D of Figure 4 colludes and increases their balance after $T_{n-k}$ of user A’s SPC, the smart contract will choose blocks from $T_{n-k}$ to $T_{n-k-V}$ uploaded by user A because the chains from the other users have invalid information between $T_{n-k}$ and $T_{n-k+1}$ despite the highest round count.

Each user stores blocks of the last SPC cycle, [n−V,n−1], at the current t=n to prove their balance; a user does not need to maintain older transactions than the n−V since it already confirms the state before n−V, and we can save local storage by removing obsolete transactions.

### 4.4. Concurrent Payments in Independent SPCs

The aforementioned sequential payments induce significant delays as the number of PSPs increases in the MPC. For example, users should wait for six SPC instances to pay again to a particular user in Figure 5. The delay of each SPC consists of the handshaking time for the SPC update and local chain propagation. Additionally, the verification time for the previous *n* blocks is required, but it is negligible compared to the communication delay. We denote the delay of each SPC as Δ in this study.

To reduce such a turnaround delay, we form a group of independent SPCs for concurrent payments free from double spending. In Figure 4, payments on the SPC $v_{AB}$ and $v_{CD}$ can be conducted simultaneously because each SPC does not interfere with the other concerning the MPC state. The parallel SPCs can reduce the turnaround time from 6Δ to 3Δ.

**Figure 6:**
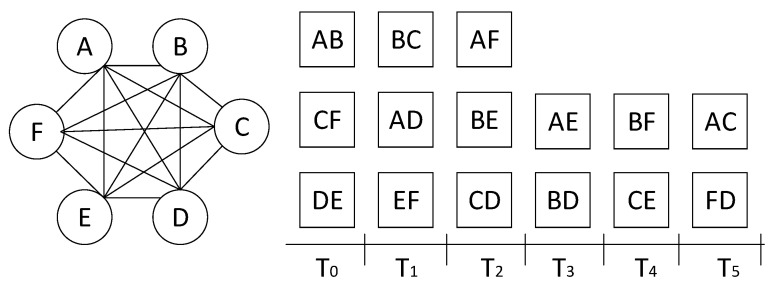
Independent SPC schedule for concurrent transactions in a 6-party MPC.

Figure 6 illustrates an example of concurrent payments on independent SPCs of the six-party MPC. The figure shows that the MPC has the first several rounds with a maximum independent set of *N*/2 = 3 SPCs and k=2 SPCs in the remaining rounds. There is an optimal SPC schedule only with the maximum independent set during five rounds for this example. However, finding the maximum independent sets is known as NP-hard.

Therefore, we propose an iterative greedy approximation algorithm for a bounded degree graph (an approximation ratio of ($deg(v_{ij})$ + 2)/3, which finds a maximal independent set (MIS) iteratively by choosing a minimum degree SPC first at each step.

**Theorem** **1.** *The number of MISs, $M_{V}$, is bounded as proven in [49], and the number of MISs of size k, $M_{k}$, is at most [50]*

$$\begin{array}{ccc} k\leq V/3: & & \\ \;\;\;\;\alpha^{k-(V\;\mathrm{mod}\;k)}{\left(\alpha +1\right)}^{V\;\mathrm{mod}\;k}, & \;\;\;\;\;\;\;\;\alpha =\lfloor V/k\rfloor & \left(1\right)\\ V/3\leq k\leq V(V-2)/8: & & \\ \;\;\;\;3^{V/3}, & \;\;\;\;\;\;\;\;V\;\mathrm{mod}\;3=0 & \left(2\right)\\ \;\;\;\;4\cdot 3^{\lfloor V/3\rfloor -1}, & \;\;\;\;\;\;\;\;V\;\mathrm{mod}\;3=1 & \left(3\right)\\ \;\;\;\;2\cdot 3^{\left\lfloor V/3\right\rfloor }, & \;\;\;\;\;\;\;\;V\;\mathrm{mod}\;3=2 & \left(4\right) \end{array}$$

**Corollary** **1.** *The SPC cycle, r, as a minimum round for all SPCs, can be derived from the average number of simultaneous SPCs, $\sum_{k}^{N/2}k\;P\left(k,V\right)$, per round, where the probability $P\left(k,V\right)=M_{k}/M_{V}$ of the k MISs is given by Equations (1)–(4).*

(5) $$\arg\min_{r}\sum_{T=0}^{r}\sum_{k=1}^{N/2}k\;P\left(k,V\right)\geq V$$

Table 3 shows the theoretical upper bound of the average SPCs and rounds for different sizes of the MPC derived by Equation (5). The average number of SPCs converges into the maximum independent set N/2 as the probability of finding larger *k* MISs increases according to the number of users.

We implemented the SPC scheduling algorithm with the iterative MIS search described in Algorithm 3 and evaluated it with the same MPCs for comparison, as shown in Table 3; the algorithm can find the optimal schedule of N−1 rounds for each MPC. The algorithm complexity can be reduced to O(VlogV) using the heap data structure and it needs to be executed only once at the beginning of MPC creation.
**Algorithm 3:** Find independent sets of SPCs in an MPC clique.1:Initialization:2:Node set N, i,j∈N3:key, ij = deg($v_{ij}$), value v(ij) = SPC $v_{ij}$4:Make a minHeap (ij, v(ij)) →*Q*5:r=06:Instruction:7:**while** True **do**8:   Set $V_{r}=\left\{\right\}$9:   Set $M_{r}=\left\{\right\}$10:   Dequeue $v_{ij}$← root(*Q*) and Heapify(*Q*)11:   **while** *Q* is not empty **do**12:     **if** $i,j\notin M_{r}$ **then**13:        Enqueue i,j→$M_{r}$14:        Enqueue $v_{ij}$→$V_{r}$15:     **else**16:        Enqueue $v_{ij}$→$Q^{\prime }$17:     **end if**18:     Dequeue $v_{ij}$← root(*Q*) and Heapify(*Q*)19:   **end while**20:   **if** $Q^{\prime }$ is not empty **then**21:     *r* = *r* + 122:     $Q=Q^{\prime }$ and $Q^{\prime }$23:     Make a minHeap (ij, v(ij)), $ij\in Q^{\prime }$→*Q*24:   **else**25:     break26:   **end if**27:**end while**28:return $M_{r}$, *r* = [0, N − 1]

For this MPC with parallel SPCs, either full or partial synchronization can be considered, similar to the linear MPC model. The full synchronization keeps users updating an MPC state from the previous round. Meanwhile, partial synchronization has an update delay for the final status at the cost of reducing the communication overhead. For example, A can finalize the $T_{0}$ status at $T_{2}$ as it receives $v_{DE}$ from D at $T_{1}$ and $v_{CF}$ from F at $T_{2}$ in Figure 6. Although this synchronization delay increases by log-scale logV/2, owing to the accumulated blocks in the local chain, a user can suffer from communication overhead to trace the balance of a counterparty. For dispute resolution in the concurrent SPCs, the smart contract also receives all users’ previous *V* blocks (almost [n−r,n−1] rounds). It investigates each round for update as in Algorithm 2. In contrast to the linear MPC, each round is subjected to an MPC update only when all SPC transactions of a round are verified successfully.

## 5. Coalition Formation Game for MPC Creation

Although the MPC as a payment service consortium contributes to reducing the PSPs’ collateral cost, the induced payment latency is considerable according to the MPC size; accordingly, the coalitional gain is not super-additive but limited by the latency cost. Therefore, we propose the MPC creation mechanism in the coalition formation game (CFG) framework, in which independent and rational PSPs form MPCs dynamically based on the distributed formation algorithm, which leads to a stable state for the MPC partitions.

### 5.1. CFG Model

The hedonic CFG is a coalitional game that satisfies the following two hedonic conditions. In this study, the PSPs can join and leave any MPC freely according to their preference, e.g., collateral cost reduction for the MPC coalition formation. Such a gain is decided only by the MPC in their participation.

**Definition** **1.** *(Hedonic Conditions): A CFG is classified as hedonic if (i) the payoff of any player depends solely on the coalition members to which the player belongs; (ii) the coalitions are partitioned according to players’ preferences over coalitions.*

For the mathematical model for MPC creation, we first define the notions of the coalition structure and transferable utility based on the CFG theory [51,52,53].

**Definition** **2.** *A set of players in this CFG is a set of directional PCs in a PCN, L, which form a cooperative MPC coalition as a subset, $S_{k}\subseteq L$. However, the actual players are PSPs, who determine coalitions for their directional PCs by executing the formation algorithm. Therefore, the disjoint coalitions constitute the coalition structure, $\Pi =\{S_{1},S_{2},\ldots S_{K}\}$, $L=\cup_{k=1}^{K}S_{k}$, which can be denoted by a set of V, $\left\{V_{1},V_{2},\ldots ,V_{K}\right\}$. The transferable utility value $\upsilon (S_{k})$ of the coalition is determined solely by the coalition members in the characteristic form of this CFG, and it is fairly divided by SPCs.*

To derive the aggregate utility of the coalition, we address the revenue and cost of the MPC as below.

- Revenue: sum of transaction fee over PBT flows served by an MPC coalition, $\upsilon_{r}\left(S_{k}\right)$.
- Cost: deposit sum of PSPs of an MPC, $\upsilon_{c}\left(S_{k}\right)$. The collateral cost of individual PSPs is reduced by deposit sharing among multiple SPCs, which is limited by increasing payment delays from the SPC turnaround. Such an additional delay to the legacy PC, $E\left(D_{ij}^{MPC}-D_{ij}^{PC}\right),ij\in S_{k}$, demands a larger deposit, thereby increasing the collateral cost. Furthermore, the delay cost will be −∞ if it exceeds the $T_{exp}$, which can be a constraint for coalition formation.
- Utility: with the above revenue and cost, the net payoff of the coalition formation can be $\upsilon \left(S_{k}\right)=\upsilon_{r}\left(S_{k}\right)-\upsilon_{c}\left(S_{k}\right)$.

### 5.2. Coalition Revenue Function

When a PBT flow, *u*, goes through an MPC, its ingress PSP *i* and egress PSP *j* receive an incentive for each transaction [34]. Therefore, the total transaction fee can be calculated by the PBT flow rate with the transaction fee rate, $\epsilon_{r}$.

(6) $$\upsilon_{r}\left(S_{k}\right)=\sum_{i}^{N}\sum_{u}^{U}\epsilon_{r}x_{ij}^{u},$$

where the $x_{ij}$ is the PBT rate of a PC (i,j).

### 5.3. Coalition Cost Function

If each PC needs deposit amount $C_{ij}$ to afford the given PBT demand, $\sum_{u}x_{ij}^{u}\leq C_{ij}$, the total required deposit of the MPC coalition $S_{k}$ can be as below:

(7) $$\upsilon_{c}^{1}\left(S_{k}\right)=\sum_{ij\in \tilde{S_{k}}}\alpha_{ij}C_{ij},\;\tilde{S_{k}}\subseteq S_{k},$$

where $\alpha_{ij}\in \left\{0,1\right\}$ according to the existence of a PC ij, and $\tilde{S_{k}}$ is a subset of PCs with the maximum deposit for each PSP *i*, $\tilde{S_{k}}=\cup_{i}^{N}{\tilde{S_{k}}}^{i},{\tilde{S_{k}}}^{i}=\left\{ij|C_{ij}\geq C_{ih},ij,ih\in S_{k}\right\}$. For each PSP, the maximum PBT rate deposit amount can cover all SPC transactions in its SPCs.

The payment delay of the legacy PC, $D_{ij}^{PC}$, consists of transaction processing, propagation, and queuing. The transaction process delay is negligible compared to the two other factors, e.g., several micro-seconds. The message propagation delay, $D_{m,ij}^{u}$, depends on the distance and congestion in the underlying network, defined as a random variable of the exponential distribution in this study, $P\left(D_{m,ij}^{u}<t\right)=1-e^{-\Delta_{ij}^{-1}t}$, with average $E\left(D_{m}\right)=\Delta_{ij}$, as Ethernet based on carrier-sense multiple access with collision detection (CSMA/CD) has an access delay according to the binary back-off window [54] (i.e., the network delay can be modeled also by normal or Pareto distributions). Meanwhile, the queuing delay is critical according to bidirectional PBT flows modeled by M/M/1.

(8) $$D_{q_{s},ij}^{u}\left(x_{ij}^{u}\right)=\frac{\sum_{u\in U}x_{ij}^{u}}{\sum_{u\in U}x_{ji}^{u}\left(\sum_{u\in U}x_{ji}^{u}-\sum_{u\in U}x_{ij}^{u}\right)}$$

Although the queuing delay is theoretically infinite for $\sum_{u}x_{ji}^{u}<\sum_{u}x_{ij}^{u}$, the maximum delay is the lock time in the off-chain system as all transactions over $T_{exp}$ are canceled automatically. Furthermore, PSPs can cancel in-flight lock transactions using a rollback procedure in advance. For $\sum_{u}x_{ji}^{u}=\sum_{u}x_{ij}^{u}$, in which the same amount moves back and forth for bidirectional PBTs, the queuing delay of a PBT is limited by the PBT interval, $x_{ji}^{-1}$.

Moreover, the MPC delay $D_{ij}^{MPC}$ is mainly caused by the queuing and SPC turnaround. The queuing delay of the MPC, $D_{q,ij}^{u}$, is determined by the balance of the aggregated flow rate. The egress aggregated PBT flow rate from a PSP *i* to any PSP *j*, $\lambda_{ij}$ can be written as below:

(9) $$\begin{array}{cc} \lambda_{ij} & =\sum_{u\in U}\sum_{j\in N\backslash i}x_{ij}^{u}\left(1+\Sigma_{D}\right), \end{array}$$

where the maximum delay for SPC turnaround, $\Sigma_{D}=\max_{\Delta }\sum_{r=0}^{N-1}\Delta_{ij}^{r}$, depends on the transaction process and propagation delay of each SPC.

**Property** **1.** *The MPC is balanced regardless of the individual PBT flow rate if the ingress and egress flow rate is equivalent at the outlink PCs of the MPC.*

Suppose that each PSP has multiple PCs outside of the MPC, ik∈L\V, and asymmetric and bidirectional PBT flows $x_{ik}^{u}$ and $x_{ki}^{u}$ are passed through the MPC; the aggregated PBT flow from the PSP *i* to others *j* in the MPC, $\lambda_{ij}$ in Equation (9), is equivalent to $\sum_{k\in I\backslash N}\sum_{u}x_{ki}^{u}$ and $\mu_{ij}$ is the same as $\sum_{k\in I\backslash N}\sum_{u}x_{ik}^{u}$, the sum of all PBT flows toward the PSP *i*. Consequently, $\lambda_{ij}=\mu_{ij}$ for a PSP *i* as $\sum_{k\in I\backslash N}\sum_{u}x_{ki}^{u}=\sum_{k\in I\backslash N}\sum_{u}x_{ik}^{u}$ is satisfied.

**Property** **2.** *The MPC queuing delay is limited by the SPC turnaround time if the MPC is balanced, $\lambda_{ij}=\mu_{ij}$.*

Once the deposit of a particular PSP is unbalanced due to a pair of asymmetric flows, $x_{i\hat{k}}^{u}$ and $x_{\hat{k}i}^{u}$, it will be rebalanced by another pair of flows, $x_{i\bar{k}}^{u}$ and $x_{\bar{k}i}^{u}$, within the $\Sigma_{D}$ because $\lambda_{ij}=\mu_{ij}$ is guaranteed. For example, if a flow $x_{i\hat{k}}^{u}$ depletes the balance of the PSP *i* at $T_{0}$ and a new payment, $A_{i}\left(t_{m}\right)$, arrives at the next SPC $T_{1}$, the payment needs to wait for $\Sigma_{D}$ until the deposit of the PSP *i* can be $C_{ij}+A_{i}\left(T_{1}\right)$ at $T_{N}$, where $C_{ij}$ is the initial deposit of the PSP *i*. We need an effective SPC scheduling algorithm for balance stability to reduce deposit variance.

**Property** **3.** *The MPC deposit of a PSP i is $C_{ij}\left(1+\Sigma_{D}\right)$, where the $C_{ij}$ is the maximum initial deposit among coalitional PCs ij of the PSP i.*

If a legacy PC ij needs a maximum $C_{ij}$ deposit for the highest PBT flow rate, the SPC ij requires an additional deposit $C_{ij}\Sigma_{D}$ for queued transactions during the turnaround time. Accordingly, other SPCs are affordable, with less deposit for new and queued transactions than the maximum SPC.

The collateral cost for additional transaction delays due to the SPC turnaround time as $E\left(D_{ij}^{MPC}-D_{ij}^{PC}\right)≃\Sigma_{D}-E\Delta$ is as below.

(10) $$\upsilon_{c}^{2}\left(S_{k}\right)=\left\{\begin{array}{cc} \sum_{ij\in \tilde{S_{k}}}C_{ij}\left(\Sigma_{D}-E\Delta \right), & \mathrm{if}\;\Sigma_{D}\leq T_{exp},\\ -\infty , & \mathrm{otherwise}, \end{array}\right.$$

where $\tilde{S_{k}}$ is defined in Equation (7) and all PSPs have equivalent PBT flows, $\lambda_{ij}=\mu_{ij}$, within the MPC coalition, $S_{k}$. Consequently, we define the MPC CFG cost function, $\upsilon_{c}\left(S_{k}\right)=\epsilon_{c}\left(\upsilon_{c}^{1}\left(S_{k}\right)+\upsilon_{c}^{2}\left(S_{k}\right)\right)$, with an interest rate, $\epsilon_{c}$, that is comparable with the transaction fee.

### 5.4. MPC Formation Algorithm

In this CFG, the PSPs constitute multiple independent and disjoint coalitions considering deposit gains from cooperation and additional costs from the MPC size. This partition problem for the coalition structure Π is known as NP-hard, as the number of the coalitions is the Bell number. Moreover, the PCN is a fully distributed system in which a PSP joins or leaves freely through the main blockchain. This study introduces a distributed algorithm using the switch operation based on preference order.

The preference order, ${⪰}_{i}$, provides each PSP with a strict rule of preference between two coalitions during dynamic coalition formation. For instance, the relation $S_{1}{⪰}_{i}S_{2}$ indicates that a player *i* prefers to join the $S_{1}$ coalition rather than the other, $u_{i}\left(S_{1}\right)\geq u_{i}\left(S_{2}\right)$, $S_{1},S_{2}\in \Pi$.

Although revenue and cost are given to each directional PC, ij∈L, the bidirectional PCs, ij and ji, need to derive a common strategy for switching operations regardless of individual payoff. Otherwise, the individual payoff will be practically zero as the PC is not established. Therefore, the switch operation splits and merges two PCs ij and ji together between MPCs at each step. For simplicity of notation, we use *i* of ${⪰}_{i}$ for an SPC iinV hereafter.

For the preference order of an SPC *i*, $u_{i}\left(S_{k}\right)$ is the sum of individual SPC payoff without coalition and the expected coalition gain that is calculated by dividing fairly the marginal utility of the coalition $S_{k}$. Here, the fair division encourages a PSP with fewer PBT flows to join an MPC with many PBT flows.

(11) $$u_{i}\left(S_{k}\right)=\left\{\begin{array}{cc} 0, & \mathrm{if}\;S_{k}\in H_{i}\left(k\right)\\ -\infty , & \mathrm{if}\;\upsilon_{c}\left(S_{k}\right)=-\infty \\ \upsilon \left(\left\{i,\tilde{i}\right\}\right)+\frac{2(\upsilon \left(S_{k}\right)-\sum_{j\in S_{k}}^{\;}\upsilon \left(\left\{j\right\}\right))}{|S_{k}|}, & \mathrm{otherwise}, \end{array}\right.$$

where the *i* and $\tilde{i}$ are an SPC ij,ji, respectively, and $H_{i}\left(k\right)$ is a history set that contains all previous coalitions that the SPC *i* joined across all past partitions, $\Pi_{0},\Pi_{1},\ldots \Pi_{k}$.

In this study, we introduce two preference rules, Pareto and selfish order, for forming MPC coalitions. Utilitarian order, which considers social welfare altruistically, is inappropriate for PSPs to pursue their profits, i.e., payment fees and incentives for their deposits.

**Definition** **3.** *(Pareto order): Pareto order is one of the traditional preference relations for the CFG, which has strong restrictions, preventing damage for original and new coalitions, as in the following equation.*

(12) $$\begin{array}{cc} \; & S_{1}{⪰}_{i}S_{2}\Leftrightarrow \\ \; & u_{i}\left(S_{1}\right)>u_{i}\left(S_{2}\right)\;\wedge u_{j}\left(S_{1}\right)>u_{j}\left(S_{1}\backslash \left\{i\right\}\right)\\ \; & \wedge u_{k}\left(S_{2}\right)<u_{k}\left(S_{2}\backslash \left\{i\right\}\right),\forall j\in S_{1}\backslash \left\{i\right\},\forall k\in S_{2}\backslash \left\{i\right\} \end{array}$$

**Definition** **4.** *(Selfish order): In contrast to the Pareto order, a player switches coalitions only based on its expected utility in a new coalition if existing members accept. The existing members of the new MPC can reject the join request from the player if their payoff decreases with his participation.*

(13) $$\begin{array}{cc} \; & S_{1}{⪰}_{i}S_{2}\Leftrightarrow \\ \; & u_{i}\left(S_{1}\right)\geq u_{i}\left(S_{2}\right)\;\wedge u_{j}\left(S_{1}\right)\geq u_{j}\left(S_{1}\backslash \left\{i\right\}\right),\forall j\in S_{1}\backslash \left\{i\right\} \end{array}$$

**Definition** **5.** *(Switch operation): A PSP {i} as a player leaves a coalition $S_{k}$ and joins another coalition $S_{l}$ according to the preference order, denoted as $\varphi_{k,l}\left(i\right):S_{k}\backslash \left\{i\right\},S_{l}\cup \left\{i\right\}$, which changes MPC coalition formation in a PCN from initial partition $\Pi_{0},\Pi_{1},\ldots ,\Pi_{n}$ sequentially. The switching gain, $g(\varphi_{k,l}\left(i\right))$, is an individual payoff increment that can vary according to the preference order.*

**Theorem** **2.** *(Nash-stable coalition): A coalition Π is Nash-stable if and only if no player individually improves its utility via the switch operation. $g(\varphi_{k,l}\left(i\right))\leq 0$ for all $S_{k},S_{l}\in \Pi$. The selfish and Pareto orders enable MPC coalitions to converge into the Nash-stable coalition partitions.*

**Proof.**  Suppose that a final partition $\Pi_{f}$ from finite iterations is not Nash-stable; any PSP can perform further switch operations for higher utility, which means that the $\Pi_{f}$ is not the final coalition. Accordingly, the final partition state will be stable. Moreover, the number of switching iterations is finite, less than several possible partitions, since a player switches only to a coalition that has not been visited before.    □

Algorithm 4 shows an MPC formation function for a PSP. The PSP starts with a single-tone coalition and merges with another SPC based on preference until the Nash-stable coalition formation is established. The new MPC coalitions provide the maximum gain for each SPC in every iteration. Each SPC considers candidate MPC coalitions that have not been visited before and contain other SPCs of the same PSP to maximize their payoff. These partition updates continue until no SPC improves its payoff for further switching.
**Algorithm 4:** Distributed MPC coalition algorithm for a PSP.1:Initialization:2:User demand of PBTs, $x^{u},u\in U$ by routing3:Set PCs, $\alpha_{ij}=1$, ij∈L4:Set deposit, $C_{ij}$ for max($\sum_{u}x_{ij}^{u}$, $\sum_{u}x_{ji}^{u}$)5:Set preference order, $g(\varphi_{k,l}\left(ij\right))$6:Instruction:7:Set $S_{k}=\left\{ij,ji\right\},\forall ij$ for a PSP *i*8:Calculate utility, $u_{ij}\left(S_{k}\right)$9:**while** ij and $g(\varphi_{k,l}\left(ij\right))>0$, ij∈L **do**10:   Find $S_{k}$, $ij\in S_{k}$11:   maxGain = 012:   **for** $S_{l}\in \Pi$ and $ik\in S_{l}$ **do**13:     **if** $S_{k}\;\mathrm{not}\in H_{ij}\left(k\right)$ and $g(\varphi_{k,l}\left(ij\right))>$ maxGain **then**14:        maxGain = $g(\varphi_{k,l}\left(ij\right))$ based on Equations (11)–(13)15:        $S_{l}^{*}=S_{l}$16:     **end if**17:   **end for**18:   Request coalition join to the MPC $S_{l}^{*}$19:   Switch coalition from $S_{k}$ to $S_{l}^{*}$20:   Update Π with $S_{k}$, $S_{l}^{*}$21:**end while**22:return Π

**Figure 7:**
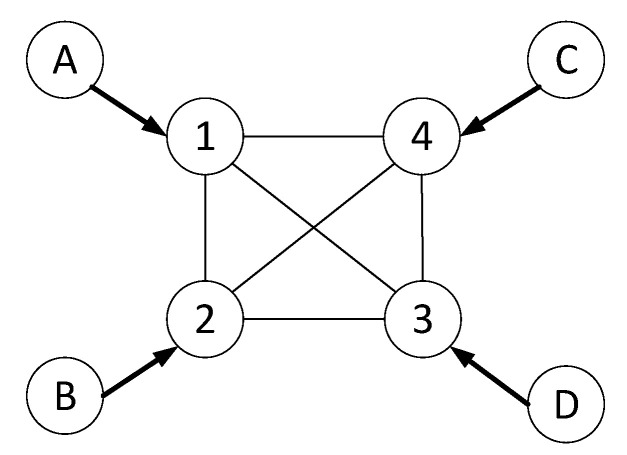
Experimental PCN topology with 4 users and a 4-party p-MPC.

We show a simple MPC CFG in Figure 7 with PBT flows demanded as in Table 4. Because all PCs are noncooperative initially, some PCs having asymmetric flows need to set a circular flow and increase the deposit amount to avoid excessive queuing delays; for instance, the imbalance of the PC $V_{12}$ by AB and BA forces PSP 1 to find a circular flow toward PSP 2 and increase the deposit up to 100 tokens. Therefore, the final deposit is 100 tokens for each PC, and 300 tokens are necessary for each PSP. Alternatively, PSP B can reduce the flow BA rate to 50, which, however does not satisfy the user demand. Meanwhile, the MPC formation of PSPs 1–4 can support all flows with $100+100\Sigma_{D}$ tokens.

Based on Equations (6), (7), (10), and (11) with parameters such as $\epsilon_{r}=0.2$, $\epsilon_{c}=0.1$, and EΔ=0.01 ($\Sigma_{D}=NE\Delta$), in the above example, the payoff of single-tone sets, such as $\upsilon (S_{k}=\left\{\left(1,2\right)\right\})$ and $\upsilon (S_{k}=\left\{\left(2,1\right)\right\})$, can be $\upsilon (S_{k}=\left\{\left(1,2\right)\right\})=0$ and $\upsilon \left(S_{k}=\left\{\left(2,1\right)\right\}\right))=\epsilon_{r}\left(100\right)-\epsilon_{c}\left(100+100\times 0\right)=10$ according to asymmetric user PBT flows. Thereby, the initial payoff of the SPC is $\upsilon (S_{k}=\left\{\left(1,2\right),\left(2,1\right)\right\}))=10$.

A newly merged MPC, $S_{k}=\left\{\left(1,2\right),\left(2,1\right),\left(1,4\right),\left(4,1\right)\right\}$, can gain additional coalition utility of 4.75 and accordingly 4.75/4=1.19 is given for each PC since the MPC utility is $\upsilon \left(S_{k}\right)=\epsilon_{r}\left(250\right)-\epsilon_{c}\left(250+250\times 0.01\right)=24.75$ and the sum of the original utility of each SPC {(1,2),(2,1)} and {(1,4),(4,1)} is approximately 20. Furthermore, the MPC clique of PSPs can increase the utility; for example, $S_{k}=\left\{\left(1,2\right),\left(2,1\right),\left(1,4\right),\left(4,1\right),\left(2,4\right),\left(4,2\right)\right\}$ results in $\upsilon (S_{k})=49.4$ and the marginal payoff for each PC is 19/6=3.17; the revenue increases through deposit reuse without changing the collateral cost. The maximum cliques 1–4 in the figure can provide a marginal payoff of 4.8 to each PC.

However, the MPC utility decreases when the SPC turnaround time is $\Sigma_{D}>\frac{\upsilon_{r}-\upsilon_{c}^{1}}{\sum_{ij\in S_{k}^{\prime }}C_{ij}}+E\Delta$. For an example of cliques 1–4, the marginal payoff is almost zero with EΔ=0.24.

## 6. Experimental Results

**Figure 8:**
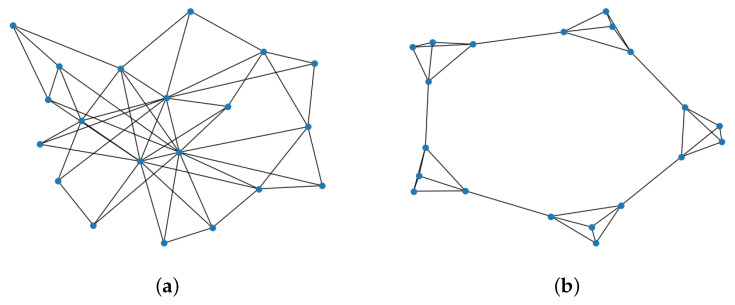
SNS-like PCN models. (**a**) Power-law cluster with 20 PSPs. (**b**) Ring of 5 cliques with 4 PSPs each.

We generate multi-PBT flows in SNS-driven PCNs and build MPC formation using the proposed CFG algorithm. Random graphs such as a power-law clustering or partitioning model with a default of 20 PSPs are used for our experiment, as shown in Figure 8. The power-law clustering has a degree of a PSP as a vertex of 3 and clique probability of 0.7 as a default. The revenue and cost rate are configured as $\epsilon_{r}$ = 0.2 and $\epsilon_{c}$= 0.1. Default EΔ is 0.1. User demand is given randomly by 50 or 100. We derive the average throughput with 100 randomly generated PCN topologies.

**Figure 9:**
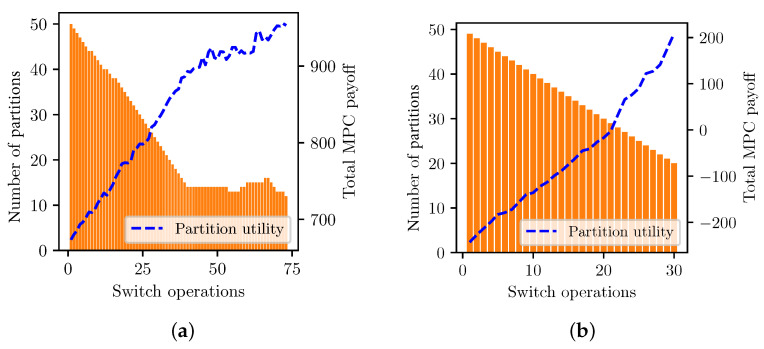
MPC partition convergence and utility. (**a**) Selfish order. (**b**) Pareto order.

First, we investigate the convergence of MPC partitioning via our distributed coalition algorithm with two preference rules. Figure 9 shows that the split and merge algorithm allows the MPCs to converge to a Nash-stable partition regarding the overall utility saturation of the PCN. For each Pareto and selfish preference order, the MPC partitions converge successfully to the equilibrium partition in very limited switch operations, 75 and 30, respectively. The selfish order constitutes an aggressive MPC, as Figure 9a shows a drastic decrement in MPCs compared to the Pareto in Figure 9b. The overall utility of the Pareto order increases monotonically. In contrast, the selfish order shows variations in several iterations because the Pareto rule prevents the payoff loss of MPCs caused by new partitions. For the Pareto test, we configure $\epsilon_{c}=0.2$, the same as $\epsilon_{r}$, for an SPC to leave an MPC without harming the payoff of other members.

**Figure 10:**
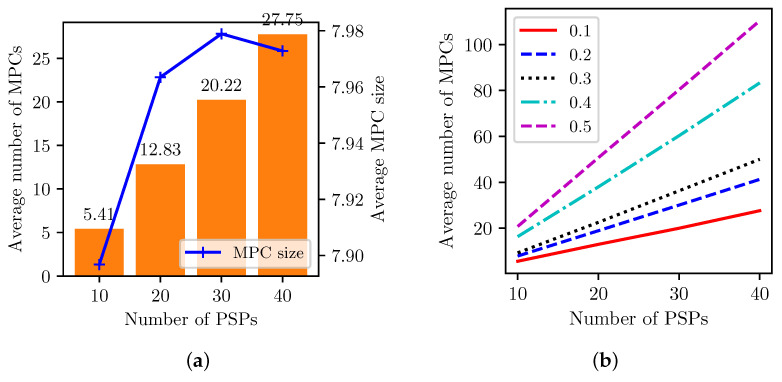
MPC formation with varying PSPs and EΔ. (**a**) Average number of MPCs and their size. (**b**) Number of MPCs with varying EΔ = 0.1∼0.5.

Figure 10a shows the average number of MPCs with varying PCN sizes regarding the number of PSPs where the selfish order was applied. The average number of MPCs almost linearly increases with more PSPs, but each size is at saturated around 8 as the degree of each PSP is limited, as in the graph topology. Figure 10b shows that the MPC formation is rather affected by the SPC turnaround delay cost, EΔ. The number of MPCs increases with the higher EΔ values; the increment is notably between 0.3 and 0.4. With 0.1, 40 PSPs only have 27 MPCs, while almost 120 MPCs have 0.5. The higher turnaround delay limits the MPC size and causes more partitions in the PCN.

**Figure 11:**
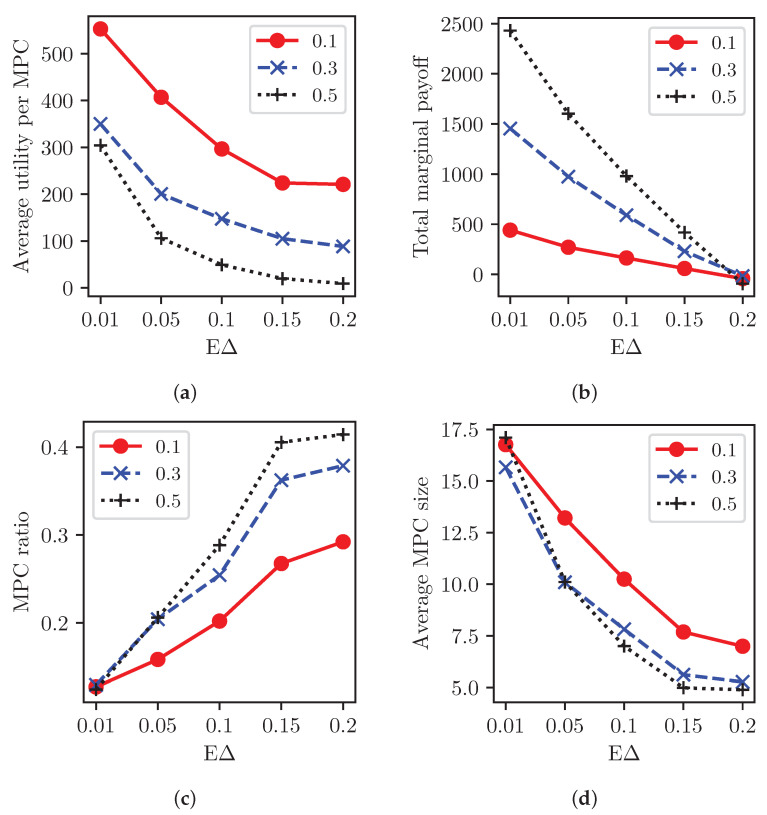
MPC formation with varying collateral cost $\epsilon_{c}=$0.1, 0.3, and 0.5 and delay cost Δ at the revenue rate $\epsilon_{r}=0.5$. (**a**) Average payoff of an MPC. (**b**) Total marginal payoff of all MPC partitions. (**c**) MPC ratio with varying cost. (**d**) Average MPC size.

In addition to the MPC delay, EΔ, collateral cost rate $\epsilon_{c}$ is critical for MPC formation at a given revenue rate $\epsilon_{r}=0.5$, as in Figure 11. Figure 11a shows the average payoff of each MPC with different $\epsilon_{c}$, which decreases exponentially with the increasing delay cost and is saturated between 0.15 and 0.2 of EΔ since there is no major change in partitions. The average utility of an individual MPC is higher with a lower cost rate $\epsilon_{c}$—for instance, 100 vs. 400 for $\epsilon_{c}=$0.5 and 0.1, respectively, at EΔ = 0.05.

Contrarily, the total marginal payoff of MPCs as the sum of all coalitional gains, $\sum_{k}\upsilon \left(S_{k}\right)-\sum_{ij}\upsilon \left(\left\{\left(i,j\right)\right\}\right)$, is higher with the cost rate $\epsilon_{c}=0.5$ than others, owing to more deposit reuse. For instance, the marginal gain increases from 500 to 2500 with $\epsilon_{c}=$0.1 and 0.5, respectively, in Figure 11b when EΔ is 0.01. However, the EΔ limits such a coalition gain.

Figure 11c shows the average MPC ratio (i.e., number of MPCs/number of initial partitions) increasing with higher cost rates, discouraging PSPs from joining the MPCs. The higher collateral cost rate aggravates the coalition gain and the turnaround delay. Accordingly, the higher $\epsilon_{c}$ generates more MPCs with small sizes (the minimum MPC size is 2 for a single SPC). For instance, $\epsilon_{c}=0.1$ merges the initial coalitions into 20% of them, while $\epsilon_{c}=0.5$ achieves only 30% in the case of EΔ = 0.1. Moreover, the number of final partitions increases as the MPC delay cost EΔ becomes significant. In Figure 11d, accordingly, each MPC size decreases with the increasing delay cost, especially for the higher cost rate $\epsilon_{c}$. The MPC ratio and size are comparable between EΔ = 0.15 and 0.2 because the coalitional payoff is limited.

**Figure 12:**
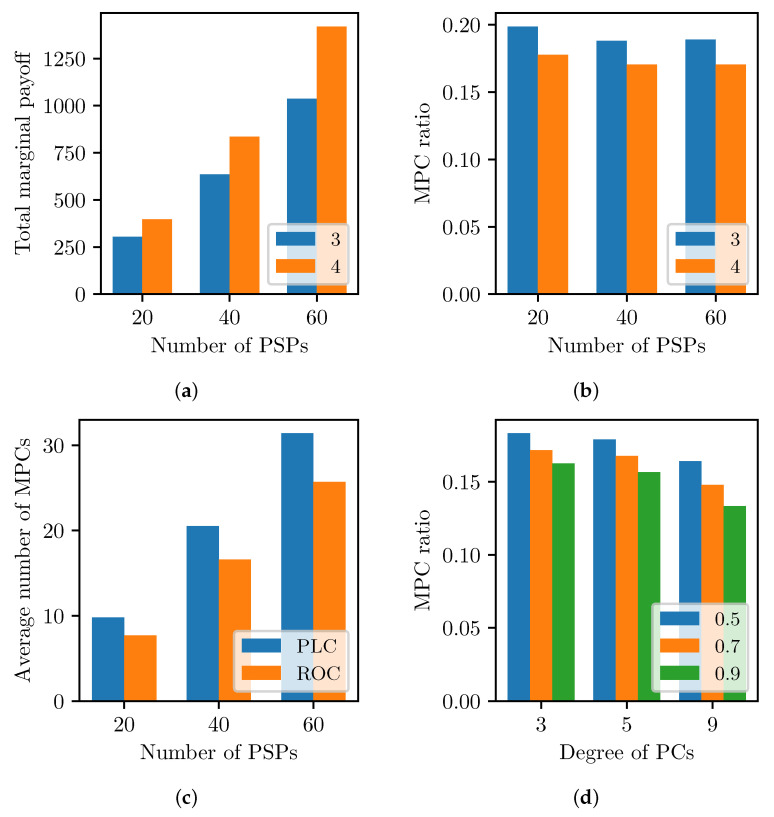
MPC formation with varying PCN topologies. (**a**) Marginal payoff with degree of PCs. (**b**) Clustering MPCs with degree of PCs. (**c**) Power-law clusters vs. ring of cliques. (**d**) MPC ratio with varying connectivity probability.

Figure 12a compares the marginal payoff when the degree of a PSP as several PCs is 3 or 4. We use the SPC delay EΔ=0.05 to reveal the impact of the degree of a PSP in the power-law cluster PCNs. The total marginal payoff increases as more PSPs participate in the coalitional game and form more MPCs, as shown in Figure 10. The result shows that PSPs with PCs probably have more opportunities to form MPCs and gain payoff. The MPC ratio with the higher degree, 4, is slightly lower than the others because PSPs can be easily merged with more PCs, as in Figure 12b. Meanwhile, the MPC ratio is almost consistent with the number of PSPs. Figure 12c describes the MPC formation performance in the two different PCN topologies shown in Figure 8. More MPCs are established in the power-law cluster graphs rather than the ring of cliques, which has MPCs that are equal to the cliques. For instance, the 40 PSP case has 10 cliques and 9 interconnecting PCs, constituting around 19 MPCs. In the random PSP-clustered network, PC existence between two PSPs increases the probability of forming MPCs, as shown in Figure 12d. The MPC ratio decreases monotonically with higher PC connectivity probability from 0.5 and 0.7 to 0.9. Moreover, a higher degree of PSP among 3, 5, and 9 helps to build larger MPCs and reduce the MPC ratio.

**Figure 13:**
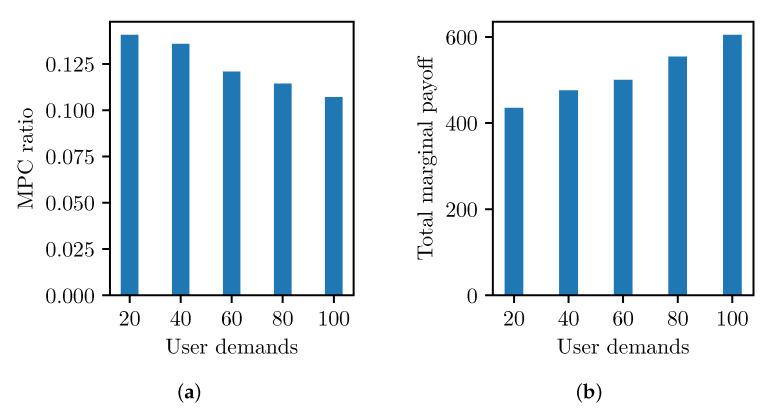
MPC formation with varying user demands. (**a**) MPC coalitions with asymmetric user demands. (**b**) Marginal utility of total partitions.

Asymmetric PBT flows of user demands also affect the MPC formation because the revenue of the MPC is proportional to the PBT flow rate. Figure 13 shows the relation of MPC formation and user demand, where we apply EΔ=0.01 to reduce the MPC overhead and highlight the impact of the user demand. Figure 13a shows that the MPC ratio decreases as the PSPs form larger coalitions for more revenue. In Figure 13b, the total marginal payoff from MPCs increases with more user demand. Consequently, more PBT flows increase the MPC utility and form a larger MPC in a profitable situation with a low MPC delay cost.

## 7. Conclusions

This study introduces the MPC scheme based on the public blockchain system with smart contract algorithms for channel management, which provides a secure PC among multiple participants with a single deposit from each PSP. This proposed idea enables users to reduce the collateral cost for multiple separate PCs for other users and also the risk of channel depletion due to asymmetric PBT flows.

Furthermore, we propose a distributed MPC formation algorithm for each PSP, adopting a CFG framework. This is the first study to propose a formation algorithm for MPC creation. The MPCs are established by consecutive switching operations considering the marginal utility between the payoff from the shared deposit and the delay cost induced by SPC turns.

Experimental results show that the proposed algorithm successfully establishes MPC coalitions with marginal gains. Their sizes depend on the SPC turnaround delay and collateral cost, rather than the PCN size and degree of SPCs. However, marginal utility by the MPC formation increases with a larger number of PSPs, transactions, and connectivity among PSPs.

In this study, we demonstrate theoretically the feasibility of the MPC concept and its formation algorithm based on several network models. Therefore, we will evaluate our system using an MPC simulator implemented by NS-3 for a real network environment. In addition, we will conduct further study to reduce the SPC turnaround time for a larger MPC and more efficient scheduling algorithm of SPCs, rather than the round robin that we now use.

## Figures and Tables

**Table 1:** Previous works for imbalanced deposit in PCN.

Name	Cryptocurrency	Layer	Main Idea
Revive [35]	Any	L2	Circular PBT flow
Channel Factory [38]	Bitcoin	L1.5	Create timelock sub-transactions
Perun [39,40]	Ethereum	L2	Create virtual channel using smart contract
Perun extend [42,43]	Bitcoin	L2	Create virtual channel using timelocks and multi-signatures
Circle [36]	Ethereum	L2	Circular PBT flow by smart contract
MPC [37]	Ethereum	L3	Scheduled sub-PCs in the fixed MPC
Proposed MPC	Ethereum	L2	Partially/fully synchronized MPC with dynamic topology

**Table 2:** Symbols of payment channel network model.

Symbol	Description
I, *I*	A set of users and its size
L, *L*	A set of directional PCs and its size
*ij*, *(i,j)*	A directional PC from *i* to *j*
$K_{p}^{i},K_{s}^{i}$	A public key and private key of a user *i*
$B_{t}\left(i,j\right)$	A transaction block of a SPC (i,j) at round *t*
*A*	Transaction amount
H	Hashed transaction message
U, u	A set of PBT flows and a PBT flow (s, d)
N, *N*	A set of MPC users and its size
V, *V*	A SPC set and its size
M	A MPC state
*x*	PBT flow rate
*D*	Payment delay
*p*	A PBT path
$C_{ij}$	Initial channel (i,j) deposit
$T_{exp}$	PBT time lock duration
Δ	Transaction process and propagation delay

**Table 3:** Expected concurrent SPCs, scheduling period, and experimental result.

Number of MPC Users (N)	4	6	8	10	20	40
Average SPCs	1.3	2.43	3.56	4.66	9.85	19.93
Average rounds (*r*)	4.5	6.17	7.84	9.65	19.29	39.14
Rounds by Algorithm 3	3	5	7	9	19	39

**Table 4:** User demand of PBT flows in Figure 7.

AB	50	BA	100	CA	50	DA	50
AC	50	BC	50	CB	100	DB	50
AD	100	BD	50	CD	50	DC	100

## Data Availability

Not applicable.
